# Extra-Cerebellar Signs and Non-motor Features in Chinese Patients With Spinocerebellar Ataxia Type 3

**DOI:** 10.3389/fneur.2019.00110

**Published:** 2019-02-18

**Authors:** Xiaoqin Yuan, Ruwei Ou, Yanbing Hou, Xueping Chen, Bei Cao, Xun Hu, Huifang Shang

**Affiliations:** ^1^Department of Neurology and Rare Disease Center of West China Hospital, Sichuan University, Chengdu, China; ^2^Huaxi Biobank, West China Hospital, Sichuan University, Chengdu, China

**Keywords:** spinocerebellar ataxia type 3, SCA3, extra-cerebellar signs, non-motor symptoms, fatigue, sleep disturbances

## Abstract

**Objectives:** Our study attempted to systematically explore the prevalence of extra-cerebellar signs and non-motor symptoms, such as anxiety, depression, fatigue, excessive daytime sleepiness (EDS) and sleep disturbances in a cohort of Chinese patients with spinocerebellar ataxia type 3 (SCA3), and further investigated the correlations between non-motor symptoms and clinical characteristics in SCA3 patients.

**Methods:** This study included 68 molecular-proven SCA3 patients. Extra-cerebellar signs were evaluated with the Inventory of Non-Ataxia Symptoms (INAS). The INAS count indicated the number of non-ataxia signs in each patient. The severity of ataxia, fatigue, EDS, sleep quality, anxiety, and depression were assessed using the Scale for the assessment and rating of ataxia (SARA), Fatigue Severity Scale (FSS), Epworth Sleepiness Scale (ESS), Pittsburgh Sleep Quality Index (PSQI), Hamilton Anxiety Rating Scale (HAMA), and the Hamilton Depression Rating Scale (HAMD) (24 items), respectively.

**Results:** Extra-cerebellar signs were detected in 91.2% of all SCA3 patients and the mean total INAS count was 2.72 ± 1.88. Rigidity was the most frequent extra-cerebellar sign (47.1%, *N* = 32). Sensory symptoms (2.9%, *N* = 2) and chorea (5.9%, *N* = 4) were rare, and myoclonus (0%) was not found in this cohort. High frequencies of sleep disturbances (64.7%), fatigue (52.9%), depression (48.5%), and anxiety (42.6%) were detected in SCA3 patients. The Spearman correlation indicated that the HAMD score was associated with the CAG repeat length and HAMA score, while the PSQI score was correlated with the SARA and FSS score. In addition, multivariate linear regression analysis showed that the CAG repeat length, age of onset, sleep disturbances and depression were significant predictors of fatigue in SCA3 patients.

**Conclusions:** Our study indicates that the vast majority of SCA3 patients display extra-cerebellar signs. Except for EDS, anxiety, depression, fatigue and impaired sleep quality are present in SCA3 patients. The CAG repeat length, age of onset, sleep disturbances and depression are predictors of fatigue in SCA3 patients.

## Introduction

The spinocerebellar ataxias (SCAs) are a clinically and genetically heterogeneous group of dominantly inherited neurodegenerative disorders, which mainly affect the cerebellum and its connections (1). To date, a total of 49 subtypes of SCA, including 38 causative genes, have been identified (2). Spinocerebellar ataxia type 3 (SCA3), also known as Machado-Joseph disease (MJD), is caused by abnormal trinucleotide (CAG) expansion in the *ATXN3* gene which is located in chromosome 14q (3). SCA3 has a wide range of clinical manifestations, and cerebellar ataxia is the core symptom, including progressive dysarthria, unstable walking and hand clumsiness (4). In addition, other symptoms, including extra-cerebellar signs and non-motor symptoms, are also reported in SCA3 patients, such as parkinsonism, pyramidal signs, oculomotor deficits, dystonia, cognitive impairment, fatigue, sleep disorders, and psychiatric changes (5–9). A majority of non-ataxia symptoms appear insidiously and are often underestimated in clinical practice. Although there is still a lack of effective modifying therapy in SCA3, several non-motor symptoms can be relieved with symptomatic treatment, which can improve the quality of life in patients with SCA3. Thus, a more comprehensive study of these symptoms is important for patients' management. In recent years, several studies conducted in a European population mainly focused on extrapyramidal or movement disorders of SCAs patients (10–12). However, only a few studies comprehensively evaluated the frequency of extra-cerebellar signs and non-motor symptoms of SCA3 patients (13, 14). It is well-known that SCA3 has a distinct geographic distribution and is most prevalent in certain regions of Brazil, Portugal and China (15). Ethnic differences may lead to clinical heterogeneity.

To date, the frequency of extra-cerebellar signs and non-motor symptoms of SCA3 patients, as well as associated factors, have not systemically been investigated. Therefore, the present study had two objectives. Firstly, we attempted to systematically explore the prevalence of extra-cerebellar signs and non-motor symptoms including fatigue, excessive daytime sleepiness (EDS), sleep disturbances, and psychiatric symptoms in a cohort of Chinese patients with SCA3. Secondly, we investigated the correlations between these non-motor symptoms and the clinical characteristics in SCA3 patients.

## Patients and Methods

### Patients

The current study was approved by the Ethics Committee of the West China Hospital of Sichuan University. All subjects provided and signed informed consent forms. A total of 68 patients with clinical and molecular confirmation of SCA3 from the Department of Neurology, West China Hospital of Sichuan University, between September 2013 and December 2018, were included for this cross-sectional study. Inclusion criteria were (1) definite genetic diagnosis of SCA3, and (2) age of 16 years and older. Exclusion criteria included patients with concomitant disorders that could affect the evaluation of the Scale for the assessment and rating of ataxia (SARA) and other assessments used in the study.

Demographic data including sex, age, age of onset, and disease duration were recorded. All patients underwent a genetic test for SCA and the CAG repeat lengths were recorded. The severity of ataxia was assessed with the SARA (16). Extra-cerebellar signs were assessed with the Inventory of Non-Ataxia Symptoms (INAS) (17). INAS contains 30 items, each of which is related to one of the following 16 symptoms or signs: areflexia, hyperreflexia, extensor plantar response, spasticity, paresis, amyotrophy, fasciculations, myoclonus, rigidity, chorea, dystonia, resting tremor, sensory symptoms, brainstem oculomotor signs (horizontal and vertical ophthalmoparesis, slowing of saccades), urinary dysfunction, and cognitive impairment. In the analysis, only the presence or absence of each symptom was considered. As several INAS items were related to one symptom, the symptom was counted as present if at least one item was positive. The number of extra-cerebellar symptoms was counted in each patient and recorded as the INAS count, ranging from 0 to 16.

Fatigue was assessed with the Fatigue Severity Scale (FSS). The FSS contains nine items, and each item is scored from 1 to 7. The summed score (maximum, 63) is divided by 9, and a total score of 4 or higher indicates the symptom of fatigue (18). EDS was evaluated by using the self-administered Epworth Sleepiness Scale (ESS) (19), and a score of 10 or higher (range, 0–24) is regarded as an indicator of EDS. Quality of sleep was assessed with the Pittsburgh Sleep Quality Index (PSQI) (20), and a score of more than 5 (range, 0–21) suggests poor sleep quality. Anxiety was evaluated with the Hamilton Anxiety Rating Scale (HAMA) (21), and a score of more than 5 indicates anxiety. Depression was assessed using the Hamilton Depression Rating Scale (HAMD) (24 items) (22), and a score of 8 or higher suggests depression.

### Statistical Analysis

All continuous variables including age, age of onset, disease duration, CAG repeat length, INAS count, scores of HAMA, HAMD, ESS, PSQI, and FSS were presented as mean ± standard deviation (SD). A normality test was performed using the Kolmogorov-Smirnov test. All categorical data were presented as numbers and percentages. A Spearman correlation test was used to evaluate the relationships between non-motor symptoms and clinical characteristics. The correlation coefficient (rs) was defined as follows: ≥0.8 indicated a very strong correlation, 0.60–0.79 a strong correlation, 0.4–0.59 a moderate correlation, 0.20–0.39 a weak correlation, and ≤ 0.19 a negligible correlation. Based on the results of the Spearman correlation analysis, a multivariate linear regression analysis was used to explore the potential clinical factors related to fatigue. Any significant variables, identified by Pearson's correlation (*p* < 0.02), were further included into the multiple linear regression model. A statistical analysis was performed with the Statistical Package for the Social Sciences (SPSS) version 23.0. All statistical tests were two-tailed and *p* < 0.05 was considered statistically significant.

## Results

Demographic and clinical characteristics of the SCA3 patients are listed in Table 1. Sixty-eight SCA3 patients were included in the study. The mean age was 45.25 ± 11.61 years and the mean disease duration was 5.60 ± 5.14 years. The mean CAG repeat length was 67.13 ± 9.68 and the mean INAS count was 2.72 ± 1.88. 91.2% of SCA3 patients displayed at least one extra-cerebellar sign. Twenty-nine patients (42.6%) had anxiety and 33 patients (48.5%) had depression. The prevalence of EDS, fatigue and impaired sleep were 11.8, 52.9, and 64.7%, respectively.

**Table 1:** Clinical and demographic variables of SCA3 patients.

	**SCA3 (*N* = 68)**
Age, y	45.25 ± 11.61
Sex (male/female)	27/41
Age of onset	39.81 ± 11.79
Disease duration	5.60 ± 5.14
CAG repeat length	67.13 ± 9.68
SARA	11.01 ± 4.97
INAS count	2.72 ± 1.88
Patients with INAS, *n*	62 (91.2%)
HAMA	6.76 ± 6.61
Anxiety, *n*	29 (42.6%)
HAMD	8.75 ± 8.58
Depression, *n*	33 (48.5%)
ESS	4.31 ± 4.16
EDS, *n*	8 (11.8%)
PSQI	7.15 ± 4.63
Poor sleep, *n*	44 (64.7%)
FSS	3.78 ± 2.42
Fatigue, *n*	36 (52.9%)

*SARA, Scale for the assessment and rating of ataxia; INAS count, inventory of non-ataxia symptoms count; HAMA, Hamilton Anxiety Rating Scale; HAMD, Hamilton Depression Rating Scale; ESS, Epworth Sleepiness Scale; EDS, excessive daytime sleepiness; PSQI, Pittsburgh Sleep Quality Index; FSS, Fatigue Severity Scale*.

The frequencies of each extra-cerebellar symptom are presented in Table 2. Rigidity was the most common sign (47.1%, *N* = 32). Others were, in the order of frequency, hyperreflexia (39.7%, *N* = 27), extensor plantar (32.4%, *N* = 22), brainstem oculomotor signs (23.5%, *N* = 16), spasticity (20.6%, *N* = 14), fasciculations (19.1%, *N* = 13), urinary dysfunction (17.6%, *N* = 12), muscle atrophy and rest tremor (16.2%, *N* = 11), paresis and cognitive dysfunction (14.7%, *N* = 10), dystonia (7.4%, *N* = 5), areflexia and chorea/dyskinesia (5.9%, *N* = 4), Sensory symptoms were rare (2.9%, *N* = 2), and myoclonus was not seen in our SCA3 patients.

**Table 2:** Frequency of Extra-cerebellar signs in SCA3 patients.

**Non-ataxia signs**	**Frequency (*N*%)**
Hyperreflexia	27 (39.7%)
Areflexia	4 (5.9%)
Extensor plantar	22 (32.4%)
Spasticity	14 (20.6%)
Paresis	10 (14.7%)
Muscle atrophy	11 (16.2%)
Fasciculations	13 (19.1%)
Myoclonus	0
Rigidity	32 (47.1%)
Chorea/Dyskinesia	4 (5.9%)
Dystonia	5 (7.4%)
Resting tremor	11 (16.2%)
Sensory symptoms	2 (2.9%)
Urinary dysfunction	12 (17.6%)
Cognitive dysfunction	10 (14.7%)
Brainstem oculomotor signs	16 (23.5%)

The correlations between the scores of HAMA, HAMD, FSS, ESS, PSQI, and the clinical variables are listed in Table 3. The HAMD score was correlated with the HAMA score (rs = 0.78, *p* < 0.0001) and the CAG repeat length (rs = 0.32, *p* = 0.008). The FSS score had a relationship with the CAG repeats (rs = 0.47, *p* < 0.0001), age of onset (rs = −0.46, *p* < 0.0001), HAMD score (rs = 0.38, *p* = 0.001) and the PSQI score (rs = 0.34, *p* = 0.004). The PSQI score was associated with the SARA and FSS score. No significant relationship was found between the ESS score and the clinical variables including the CAG repeat length, age of onset, disease duration, SARA, HAMA, and the HAMD score.

**Table 3:** Correlations between non-motor symptoms and clinical variables in SCA3 patients.

	**HAMA**	**HAMD**	**ESS**	**FSS**	**PSQI**
CAG repeat length	NS	0.32^*^	NS	0.47^**^	NS
Age of onset	NS	NS	NS	−0.46^**^	NS
Disease duration	NS	NS	NS	NS	NS
INAS count	NS	NS	NS	NS	NS
SARA	NS	NS	NS	NS	0.31^*^
HAMA	/	0.78^**^	NS	NS	NS
HAMD	0.78^**^	/	NS	0.38^*^	NS
ESS	NS	NS	/	NS	NS
FSS	NS	NS	NS	/	0.34^*^
PSQI	NS	NS	NS	0.34^*^	/

*Correlation coefficients are listed*.

* *P < 0.01*,

** *P < 0.001*.

*INAS, inventory of non-ataxia symptoms count; SARA, scale for the assessment and rating of ataxia; HAMA, Hamilton Anxiety Rating Scale; HAMD, Hamilton Depression Rating Scale; ESS, Epworth Sleepiness Scale; FSS, Fatigue Severity Scale; PSQI, Pittsburgh Sleep Quality Index*.

The predictors of fatigue in SCA3 patients, analyzed by a multivariate linear regression model, are listed in Table 4, which include the CAG repeat length, age at onset, impaired sleep, the HAMD and ESS, as the independent variables. The final model showed that the CAG repeat length, age of onset, impaired sleep and the HAMD score were significant predictors.

**Table 4:** Multivariate linear regression analysis of FSS score.

	**B**	**SE**	**Beta**	***P*-value**
CAG repeat length	0.054	0.025	0.214	0.034
Age of onset	−0.062	0.020	−0.303	0.003
Poor sleep	1.129	0.488	0.224	0.024
HAMD	0.077	0.028	0.272	0.008

*Final model, adjusted R^2^ = 0.401, SE, standard error*.

*Dependent variables: CAG repeat length, Age of onset, Poor sleep, HAMD, ESS*.

## Discussion

The present study provides a comprehensive assessment of extra-cerebellar signs and non-motor symptoms including psychiatric symptoms, fatigue, EDS and sleep quality in a cohort of Chinese patients with SCA3. We demonstrated that 91.2% of SCA3 patients in our cohort presented extra-cerebellar signs, among which rigidity was the most common. SCA3 patients had high frequencies of depression (48.5%), anxiety (42.6%), fatigue (52.9%), and poor sleep quality (64.7%). In addition, the severity of depression was correlated with the CAG repeat length and anxiety. Sleep quality was associated with fatigue and severity of ataxia, but not with EDS and psychiatric conditions. Moreover, the CAG repeat length, age of onset, sleep disturbances, and depression are predictors of fatigue in SCA3 patients.

The mean INAS count was 2.30 ± 1.57 in our study, similar to the findings from a previous German study (1.1 ± 1.3) on 15 SCA3 patients (23), but was lower than that of a multi-center European study (5.2 ± 2.5) on 139 patients with SCA3 (14). The mean disease durations of SCA3 patients in our cohort and in the German one (5.60 ± 5.14 and 5.7 ± 3.5, respectively) were shorter than that of the multi-center European study (11.6 ± 5.9). A longitudinal study found that the annual increase of the INAS score in SCA3 patients was 0.30 ± 0.08 (24). Thus, such a discrepancy may be due to the different disease durations and ethnic differences of SCA3 patients.

In this study, extra-cerebellar symptoms were common in SCA3 patients (91.2%). Among the extra-cerebellar signs evaluated, rigidity (47.1%) was the most frequent sign in our series, which was higher than findings from other studies (about 10%) (12, 14). These studies reported that brainstem oculomotor signs (67.9%) were the most common in their cohort of SCA3 patients (13, 14). However, brainstem oculomotor signs were less frequent (23.5%) in our SCA3 group, which was supported by another study in Chinese SCA3 patients (25). Previous studies have shown that oculomotor alterations could be detected in the preclinical stage of SCAs (26). Thus, objective tests of oculomotor signs in preclinical SCA3 patients may be useful for early therapeutic intervention. In our cohort, 7.4% of SCA3 patients presented dystonia, which was consistent with an American study (7.8%) (11). However, available estimates vary between 10 and 53% among different series (10, 12–14, 27). The exact physiopathological mechanism of SCA3-related dystonia remains unknown. Chen et al., found that the cerebellum can regulate basal ganglia activity through a short latency cerebello-thalamo-basal ganglia pathway in mice (28). A recent multimodal study indicated that dystonia in SCA3 is mainly related to structural abnormalities around the motor cortices and the thalami (27). These studies suggested that cerebellar dysfunction plays an important role in dystonia (29). Urinary dysfunction is uncommon (17.6%) in our series, similar to a Korean study and another Japanese study (13 and 16.7%, respectively) (13, bibx1). It seems that urinary symptoms are less common in Asian populations.

Other extra-cerebellar signs, such as chorea is more frequently seen in SCA17, and myoclonus is common in SCA2 (7). Whereas, myoclonus and chorea have rarely been documented in patients with SCA3 (10), which is also consistent with our findings.

As for psychiatric symptoms, we found a high frequency of depression (48.5%) in SCA3 patients, which was in line with another study (57.69%) conducted in a Chinese SCA3 group (31). It indicates that a larger proportion of SCA3 patients display depression in China than in other regions (20–34%) (32, 33). Contrary to other findings, we did not find the relationship between severity of ataxia and depression (8, 31). However, we found that the HAMD score was correlated with the CAG repeat length, which was not consistent with previous studies (8). In recent years, an increasing number of studies show that the cerebellum is involved in various aspects of non-motor symptoms. Several structural and functional neuroimaging studies demonstrate that the cerebellar contributes to the depression of SCA3 patients both at a structural level and at a functional level (34). Therefore, the neurodegenerative process in SCA3 may affect the pathophysiology of depression through the communication between the cerebellum and cortical networks (35). Few studies have investigated anxiety in SCA3 patients and no prevalence of anxiety was reported. 42.6% of SCA3 patients accompany anxiety in our cohort, which is strongly correlated with depression.

In the present study, we found that the CAG repeat length, age of onset, poor sleep and depression were independent significant predictors of fatigue in SCA3 patients. The relationship between depression and fatigue has been reported (36, 37), as well as poor sleep quality (36). Furthermore, we found significant correlations of fatigue with the CAG repeat length and age of onset, which had not been reported previously. However, we did not find an association between EDS and fatigue, which was inconsistent with other studies (37, 38). This discrepancy may be due to the lower prevalence of EDS in our cohort (11.8%) compared with that of others (37.5%). Fatigue can be caused by central fatigue (impairment of motivation) and peripheral fatigue (neuromuscular energetic abnormalities) (39). Some studies found that the cerebellum played an important role in action control and motor learning, which may be involved in the model of central fatigue (36). In addition, a previous study reported that fatigue is associated with dopaminergic dysfunction within the basal ganglia in PD patients (9). Additionally, the neuroimaging study also demonstrated that SCA3 patients had dopaminergic dysfunction (6). Therefore, the pathological mechanisms underlying central fatigue may play a more important role in the development of fatigue in SCA3. Our results imply that all related factors of fatigue should be considered, especially for those easily managed symptoms, such as depression and sleep quality.

Sleep problems have not been extensively studied in patients with SCA3 (15). In our study, we found that 64.7% of SCA3 patients had poor sleep quality, which was associated with fatigue and ataxia severity. The relationship between the severity of ataxia and poor sleep has not been reported. In our study, only 11.8% of patients had EDS, which is consistent with another Brazilian study (5). However, several previous studies showed that EDS is frequent in SCA3 with a prevalence rate ranging from 37.5 to 60%, and with a high mean ESS score of 8.64 ± 6.5 (5, 37, 38, 40, 41). This existing discrepancy may be due to shorter disease duration (5.6 vs. 9.5 years) and milder disease severity of our patients than in other studies.

The present study had several limitations. Firstly, this study had a relatively small sample size. Secondly, our study was a cross-sectional study. Whether these extra-cerebellar symptoms developed before ataxia and how they change with the progression of the disease, needs to be studied further.

## Conclusion

In conclusion, the majority of SCA3 patients in a Chinese population present extra-cerebellar signs. Rigidity is the most common extra-cerebellar sign. In addition, SCA3 patients show high frequencies of anxiety, depression, fatigue and impaired sleep quality, but not EDS in our cohort. Several factors including the CAG repeat length, age of onset, poor sleep and depression are predictors of fatigue in SCA3 patients. Future studies are needed to explore the pathophysiological mechanisms behind extra-cerebellar signs and non-motor symptoms, and to longitudinally assess changes of these symptoms in patients with SCA3.

## Author Contributions

HS planned the study. XY, RO, YH, BC, XC, XH, and HS conceived, designed, and interpreted the data. XY, RO, and YH collected the data. XY drafted the paper. HS edited the paper.

### Conflict of Interest Statement

The authors declare that the research was conducted in the absence of any commercial or financial relationships that could be construed as a potential conflict of interest.
